# p38 and JNK pathways control E-selectin-dependent extravasation of colon cancer cells by modulating miR-31 transcription

**DOI:** 10.18632/oncotarget.13779

**Published:** 2016-12-02

**Authors:** Liang Zhong, Bryan Simoneau, Jacques Huot, Martin J. Simard

**Affiliations:** ^1^ St-Patrick Research Group in Basic Oncology, CHU de Québec-Université Laval Research Centre (Hôtel-Dieu de Québec), Laval University Cancer Research Centre, Quebec City, Québec, G1R 2J6, Canada

**Keywords:** c-Fos, c-Jun, GATA2, IL-1β metastasis, endothelial cells

## Abstract

Extravasation of circulating cancer cells is a key event of metastatic dissemination that is initiated by the adhesion of cancer cells to vascular endothelial cells. It requires the interaction between adhesion receptors such as E-selectin present on endothelial cells and their ligands on cancer cells. Notably, E-selectin influences the metastatic potential of breast, bladder, gastric, pancreatic, and colorectal carcinoma as well as of leukemia and lymphoma. Here, we show that E-selectin expression induced by the pro-inflammatory cytokine IL-1β is directly and negatively regulated by miR-31. The transcription of miR-31 is activated by IL-1β. This activation depends on p38 and JNK MAP kinases, and their downstream transcription factors GATA2, c-Fos and c-Jun. The miR-31-mediated repression of E-selectin impairs the metastatic potential of colon cancer cells by decreasing their adhesion to, and migration through, the endothelium. These results highlight for the first time that microRNA mediates E-selectin-dependent extravasation of colon cancer cells.

## INTRODUCTION

The metastatic process consists of sequential interrelated steps, all of which must be completed successfully to cause a secondary tumor [1]. In particular, the adhesion of cancer cells to the endothelium of blood vessels is a prerequisite for extravasation of circulating cancer cells and their metastatic dissemination. This adhesive event requires specific interactions between adhesion receptors present on vascular endothelial cells and their ligands on cancer cells. E-selectin is an inducible member of the selectin adhesion receptor family that is expressed exclusively by endothelial cells stimulated by pro-inflammatory cytokines such as tumor necrosis factor-α (TNFα) and interleukin-1β (IL-1β) [2]. The physiological role of E-selectin is to mediate the adhesion and subsequent rolling of leukocytes on the endothelium. Thereafter, the rolling leukocytes bind more firmly to other cell adhesion molecules (ICAM and VCAM), which lead to their extravasation into inflamed tissues [3]. Cancer cells can hijack the inflammatory process and use E-selectin to extravasate and form metastases [4]. Several studies suggest that E-selectin is a key determinant of metastasis. The binding efficiency of colon cancer cell lines to E-selectin is proportional to their respective metastatic potential [5] and hence, anti-E-selectin antibodies are capable of reducing experimental liver metastasis [6]. Other cancer types showing a strong E-selectin-dependent metastatic pattern include breast, bladder, gastric, pancreatic, and colorectal carcinoma as well as some leukemia and lymphoma [2, 7]. The expression of E-selectin relies on the activation of JNK, p38 and PI3K pathways. However, apart from its degradation taking place in lysosomes [8], little is known about mechanisms that downregulate E-selectin and inhibit E-selectin-mediated extravasation.

MicroRNAs (miRNAs) are single-stranded, evolutionarily conserved, small (~21 nucleotides long) non-coding RNAs. At first, they are transcribed as primary miRNAs (pri-miRNAs), mainly from intergenic or intronic regions by RNA polymerase II. Pri-miRNAs are processed by Drosha-DGCR8 complex in the nucleus to produce precursor miRNAs (pre-miRNAs), which are exported to the cytoplasm to be cleaved by Dicer, producing mature miRNAs that are loaded into the miRNA-induced silencing complex (miRISC). Through base pairing with the 3′ untranslated region (3′ UTR) of mRNA, miRNA guides the miRISC to its target, thereby repressing translation with or without causing its degradation. Interestingly, a recent finding suggested that, the expression of E-selectin is regulated by miR-31 during inflammation [9]. It is therefore possible that miR-31 contributes to the regulation of E-selectin-dependent extravasation of cancer cells as well.

In this study, we show that IL-1β induces the expression of miR-31 in endothelial cells, which directly targets the 3′UTR of E-selectin mRNA and inhibits its expression. We also report that IL-1β-induced an increase of miR-31 expression that results from activation of both p38 and JNK MAP kinases, and their downstream transcription factors GATA2, c-Fos, and c-Jun. Specific inhibition of miR-31 is associated with increased E-selectin-dependent adhesion and transendothelial migration of colon cancer cells.

## RESULTS

### IL-1β induces the expression of miR-31, which affects E-selectin abundance

**Figure 1:**
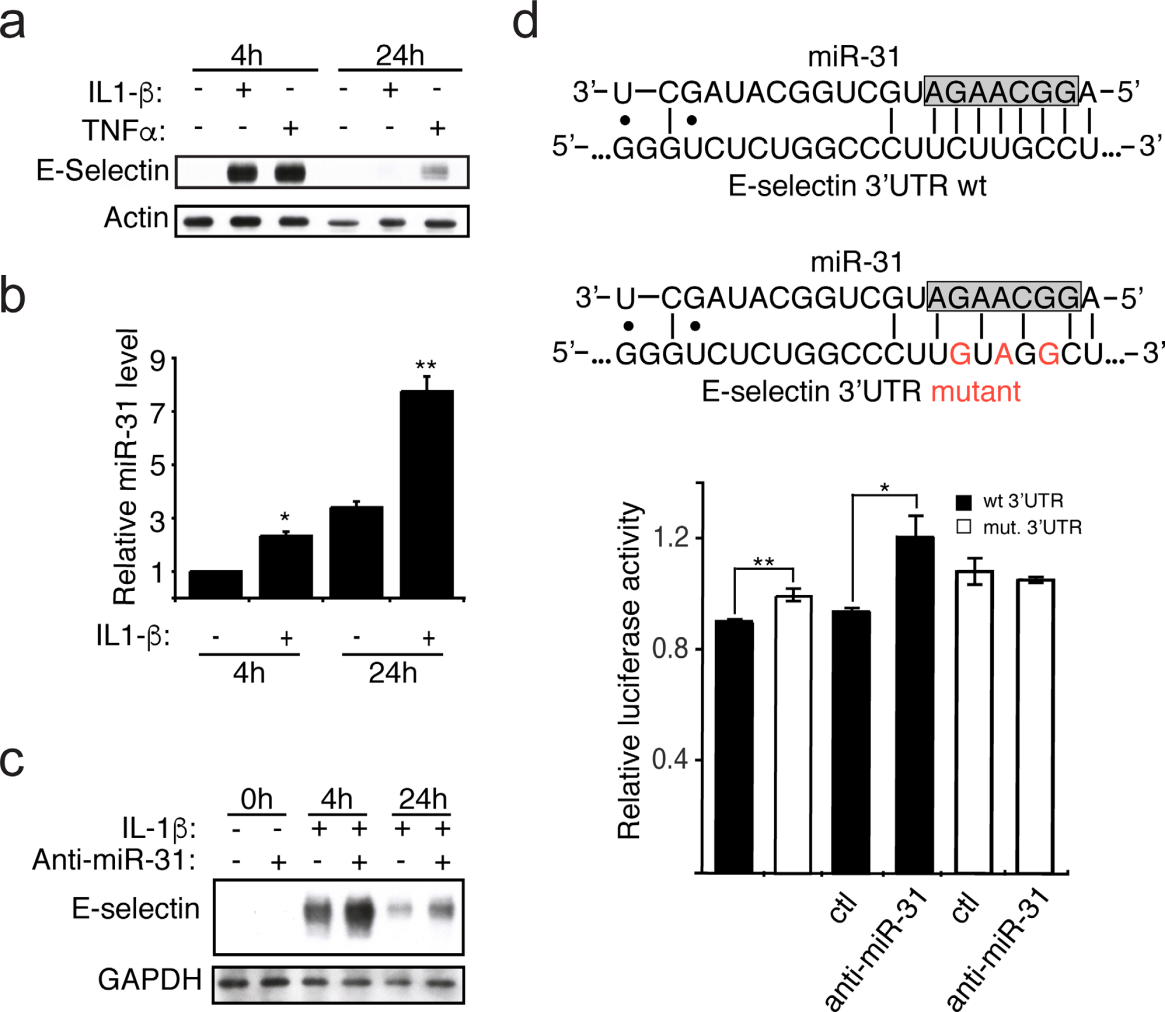
IL-1β induces the expression of miR-31, which affects E-selectin abundance **a.** The monolayer of HUVEC endothelial cells was treated with IL-1β (20ng/ml) or TNFα (10ng/ml) for indicated hours. Western blotting monitored the expression of E-selectin and Actin used as loading control. **b.** The miR-31 level was measured by quantitative reverse transcription-PCR and the snRNA U6 was used as the normalization control. **c.** Endothelial cells were transfected with 30nM of either anti-miR-31 (+) or corresponding inhibitor negative control (−) before the addition of IL-1β. The Western blots shown are representative of three independent experiments. The endogenous GAPDH was used as loading control. **d.** Top: The representation of miR-31 base-pairing with either E-selectin wild-type (wt) or mutated (mut) 3′ UTR sequence. The nucleotides in the gray box represent the seed region of miR-31, region important for target interaction. Bottom: E-selectin 3′UTR mediated reporter assays. Vector expressing luciferase reporter under the regulation of either wild-type (black bars) or mutated (white bars) E-selectin 3′UTR were transfected into HEK293T cells. 48 hours after transfection, relative luciferase activities were measured. Another set of reporters transfected cells were also transfected with 30nM of either anti-miR-31 or corresponding inhibitor negative control (ctl) to further test the contribution of miR-31 in E-selectin regulation. The error bars shown in panel b and d represent standard errors of three and six independent experiments, respectively. The significance was analyzed using a Student's *t*-test (*p <0.05; **p<0.01).

E-selectin is an adhesion receptor expressed exclusively by endothelial cells upon stimulation by inflammatory cytokines, such as TNFα and IL-1β [10]. Following exposure of human umbilical vein endothelial cells (HUVECs) to IL-1β, the expression of E-selectin was greatly induced at 4h and decreased to near null level at 24h (Figure 1a). On the other hand, the expression of miR-31 was significantly increased following exposure to IL-1β, especially at 24h, which was inversely correlated with the observed decrease of E-selectin (Figure 1b). To further confirm this inverse correlation, endothelial cells were transfected with an inhibitor of miR-31 (henceforth anti-miR-31), which clearly increased the level of E-selectin (Figure 1c). This finding was further confirmed in another type of endothelial cells (human liver sinusoidal microvascular endothelial cells or HLSMECs, Supplemental Figure 1a) even if the endogenous level of miR-31 was significantly higher in those cells (Supplemental Figure 1b). Taken together, these findings indicate that miR-31 is an important regulator of E-selectin expression in endothelial cells.

To investigate whether miR-31 directly targets E-selectin by binding to the 3′UTR of its mRNA, we carried out a dual-luciferase reporter assay. The 3′UTR of E-selectin mRNA was cloned downstream of the *Renilla* luciferase reporter gene in an expressing vector that constitutively expresses firefly luciferase. The plasmid was transfected into HEK293T cells, a cell line constitutively expressing miR-31 [11, 12]. After 48 hours, we measured the *Renilla* luciferase activity in transfected cells and observed that the *Renilla* luciferase expression under the control of the wt 3′UTR of E-selectin mRNA, was significantly lower than the cells transfected with *Renilla* luciferase reporter construct with mutations in the miR-31 binding site within E-selectin 3′UTR (Figure 1d). To further confirm the miR-31-dependent regulation of E-Selectin 3′UTR, we co-transfected these cells with either control or inhibitor of miR-31 (anti-miR-31). When assessing the *Renilla* luciferase activity, we observed that only the reporter carrying the wt E-selectin 3′UTR was significantly relieved of the repression by miR-31 (Figure 1d).

Taken together, these findings suggest that miR-31 represses the expression of E-selectin through binding to the 3′UTR of its mRNA.

### IL-1β induces the transcription of miR-31 via p38/JNK pathways

**Figure 2:**
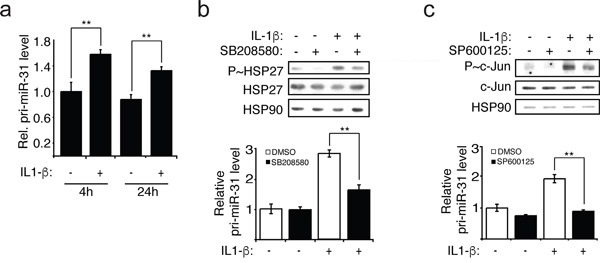
IL-1β induces the transcription of miR-31 via p38 and JNK **a.** The pri-miR-31 levels were measured relative to GAPDH mRNA using quantitative reverse transcription-PCR (qRT-PCR). **b-c.** HUVEC endothelial cells were pre-treated with 5μM of p38 inhibitor SB203580 or 10μM of JNK inhibitor SP600125 for 1 hour before the addition of IL-1β (20ng/ml). The inhibitions were confirmed by Western blotting showing decreased of phospho-HSP27 (P~HSP27) and phospho-c-Jun (P~c-Jun), downstream of p38 and JNK, respectively. The endogenous HSP90 was used as loading control. Pri-miR-31 levels relative to GAPDH mRNA were determined by qRT-PCR. The Western blots are representative of four independent experiments. The error bars represent standard errors of four independent experiments and significance was analyzed using a Student's *t*-test (*p <0.05; **p<0.01).

Since the IL-1β treatment of endothelial cells lead to an increase of the expression of miR-31, this raised the question whether the transcription of miR-31 is affected. When evaluating the level of the transcript of miR-31 gene locus called primary miR-31 or pri-miR-31, we observed an increase in its level when cells were treated with IL-1β, indicating that exposure to this cytokine induces the transcription of miR-31 (Figure 2a).

The expression of E-selectin is regulated by several signalling pathways that are activated by IL-1β, including ERK, JNK, PI3K and p38 pathways [13–15]. In order to investigate whether the activation of these pathways in response to IL-1β also regulates the transcription of miR-31, endothelial cells were treated with IL-1β in the presence or the absence of inhibitors of MEK1/2 (PD098059), JNK (SP600125), PI3K (LY294002) or p38 (SB203580). Thereafter, the activation of ERK was determined by measuring its phosphorylation whereas activities of the other three kinases were assayed by evaluating the phosphorylation of downstream targets: c-Jun (JNK), Akt (PI3K) and HSP27 (p38). The levels of pri-miR-31 and of mature miR-31 were determined concomitantly. We observed that IL-1β treatment increased the activation of each pathway: p38 and JNK (Figure 2b and 2c) as well as ERK and PI3K (Supplemental Figure 3a and 3b). The levels of pri-miR-31 and miR-31 were independent of ERK and PI3K given that they were not affected by PD098059 and LY294002 treatment, respectively (Supplemental Figure 3). In contrast, inhibiting JNK1 with SP600125, or p38 with SB203580 were associated with decreased transcription and expression of miR-31 (Figure 2b and 2c, Supplemental Figure 2). These results strongly suggest that IL-1β acts through JNK and p38 to induce the transcription of miR-31.

**Figure 3:**
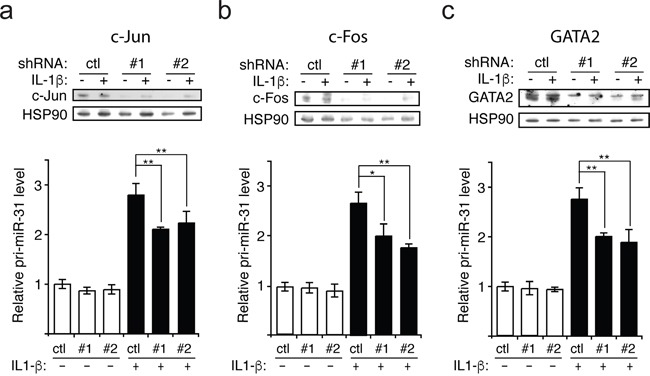
IL-1β induces the transcription of miR-31 via c-Jun, c-Fos and GATA2 **a-c**. Upon knockdown with lentivirus expressing either control (scramble shRNA) or two different shRNAs (#1 and #2) targeting each transcription factor (c-Jun, c-Fos and GATA2), the level of primary miR-31 (pri-miR-31) transcript relative to GAPDH mRNA (control) was determined by qRT-PCR. The knockdowns for each transcription factor were validated by Western blotting and HSP90 was probed and used as loading control. The Western blots are representative of three independent experiments. The error bars represent standard errors of three independent experiments and significance was analyzed using a Student's *t*-test (*p <0.05; **p<0.01).

We next investigated which transcription factors downstream of p38 and JNK are involved in regulating the transcription of miR-31. Based on the publicly available Chromatin ImmunoPrecipitation Sequencing (ChIP-seq) data from UCSC Genome Browser, we selected three transcription factors that are associated with the regulatory region of miR-31 gene that could be involved in transcribing miR-31, namely c-Jun, c-Fos and GATA2. C-Jun can be activated by JNK [16] while c-Fos and GATA2 can be activated by p38 [17–20]. To determine whether these transcription factors are involved in the production of miR-31, lentiviral vectors expressing shRNAs were employed to silence each of these transcription factors in endothelial cells, and knockdown efficacy was assessed by Western blotting (Figure 3). We observed that the knockdown of all three transcription factors significantly decreased the level of pri-miR-31 and thus, that of miR-31 (Figure 3 and Supplemental Figure 4). These data suggest that upon IL-1β treatment of endothelial cells, the activation of c-Jun mediated by JNK as well as the activation c-Fos and GATA2 by p38 lead to an increase of miR-31 expression.

### Mir-31 modulates E-selectin-mediated adhesion of colon cancer cells to endothelial cells

**Figure 4:**
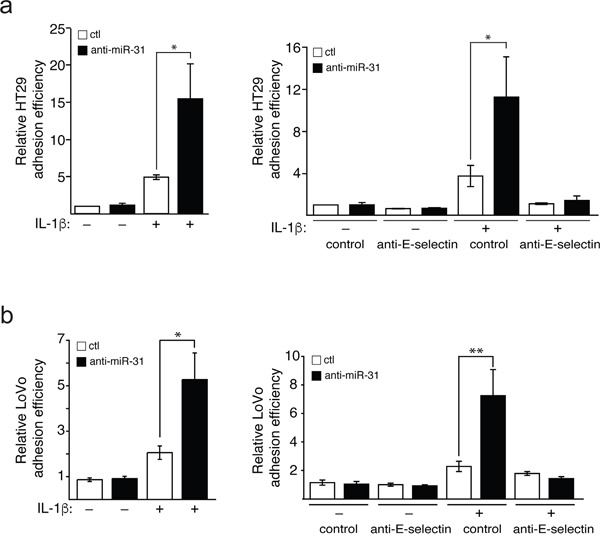
Mir-31 inhibits E-selectin-dependent adhesion of cancer cels to endothelial cells HUVEC endothelial cells were transfected and stimulated as described in Figure 1. Calcein AM-stained HT29 **a.** or LoVo **b.** metastatic cancer cells were added on a tight layer of endothelial cells and incubated for 30 minutes. Non-adhering cells were washed and those cells that adhere to endothelial cells were determined. To test the E-selectin dependence of the effect of miR-31 on cancer cells adhesion, anti-E-selectin antibody or MOPC21 antibody (used as control) was added to endothelial cells one hour before the addition of HT29 and LoVo cells (a and b respectively, right panels). The error bars represent standard errors of three independent experiments and *p* values were obtained using a Student's *t*-test (*p <0.05, **p <0.001).

Adhesion of colon cancer cells to endothelial cells expressing E-selectin is a prerequisite to their transendothelial migration (TEM) during metastatic dissemination [1, 21]. In order to examine whether miR-31 could affect the adhesion of colon cancer cells to endothelial cells, we performed adhesion assays of HT29 and LoVo metastatic colon cancer cells on endothelial cells transfected with anti-miR-31 or its control. The inhibition of miR-31 was associated with an over two-fold increase in the adhesion of both HT29 and LoVo cells compared to the control inhibitor (Figure 4a and 4b respectively, left panels). The treatment with anti-miR-31 could no longer increase the adhesion of colon cancer cells when endothelial cells were not stimulated with IL-1β and thereby did not express E-selectin (Figure 4a and 1a). Moreover, treating endothelial cells with E-selectin neutralizing antibody significantly reduced the effect of anti-miR-31 (Figure 4a and 4b, right panels), indicating that the increase in the adhesion of cancer cells mediated by inhibiting miR-31 was E-selectin-dependent. Overall, these results indicate that by regulating the expression of E-selectin, miR-31 is an important modulator of E-selectin-dependent adhesion of colon cancer cells to endothelial cells.

### MiR-31 modulates E-selectin-mediated transendothelial migration of colon cancer cells

**Figure 5:**
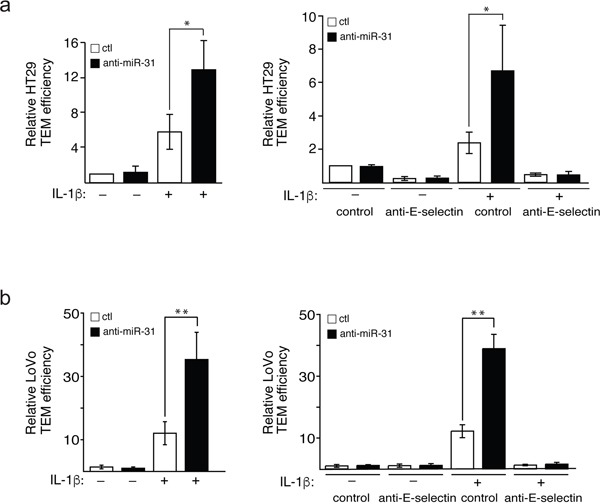
Mir-31 inhibits E-selectin-dependent transendothelial migration of cancer cells Fluorescent HT29 **a.** or LoVo **b.** cells that penetrated a tight layer of endothelial cells were counted. To test the E-selectin dependence of the effect of miR-31 on cancer cells adhesion, anti-E-selectin antibody or MOPC21 antibody (used as control) was added to endothelial cells one hour before the addition of HT29 and LoVo cells (a and b respectively, right panels). The error bars represent standard errors of three independent experiments and *p* values were obtained using a Student's *t*-test (*p <0.05, **p <0.001).

The TEM of cancer cells is associated with their motile and survival potentials [10], which are enhanced by their binding to E-selectin [22]. Since miR-31 is involved in modulating the expression of E-selectin and the adhesion of HT29 cells to endothelial cells, we next studied whether miR-31 modulates TEM of HT29 and LoVo cells by determining the capacity of these cells to cross a tight layer of endothelial cells transfected with anti-miR-31 or its control, in a Boyden chamber. We observed that the TEM of both HT29 and LoVo cells was increased by about two-fold upon inhibition of miR-31 (Figure 5a and 5b respectively, left panels). Pre-treating E-selectin expressing endothelial cells with anti-E-selectin antibody almost completely abolished the increase in TEM supporting the essential role played by E-selectin in the process (Figure 5a and 5b, right panels).

Taken together, these data are consistent with the fact that miR-31 is an important modulator of the metastatic process by targeting E-selectin.

## DISCUSSION

Several lines of evidence indicate that cancer cells hijack the inflammatory system and interact with E-selectin to extravasate and form metastases. Nevertheless, despite the importance of E-selectin in metastatic progression, little is known about the mechanisms that downregulate its expression and stop the E-selectin-mediated adhesion process. Here, we show that miR-31 is induced by IL-1β and that its expression is inversely correlated with that of E-selectin and E-selectin-mediated metastatic potential of colon cancer cells. We further report that the expression of miR-31 in response to IL-1β is stimulated at the transcriptional level in a process involving JNK and p38 MAPK pathways and their downstream transcription factors c-Jun, c-Fos and GATA 2.

The conclusion that miR-31 regulates the expression of E-selectin is supported by four independent but complementary observations. First, IL-1β-induced expression of miR-31 is inversely correlated in HUVECs with that of E-selectin. Second, inhibiting miR-31 is associated with an increase of E-selectin level both in HUVECS and HLSMECs. Lastly, altering the predicted binding site of miR-31 in the 3′UTR of E-selectin mRNA hinders its repression, supporting a direct role of miR-31 in E-selectin regulation.

An important novelty of our study is the identification of p38 and JNK as major pathways regulating the expression of miR-31. This is supported by the observation that both pathways are induced by IL-1β and that their inhibition impairs the IL-1β-induced increase in miR-31. The regulation occurs at the transcriptional level given that: (1) IL-1β increased the level of pri-miR-31 and; (2) a decreased level of miR-31 in the presence of inhibitors is accompanied by a reduction of pri-miR-31 (down to basal level without IL-1β). Chromatin ImmunoPrecipitations followed by sequencing (ChIP-seq) data from UCSC Genome Browser show that c-Jun, c-Fos and GATA2 are transcription factors bound to regulatory sites of the genome of miR-31. They can act as transcription factors regulating miR-31 following IL-1β stimulation. Along these lines, the knockdown of c-Fos, c-Jun and GATA2 in endothelial cells reduced the IL-1β-induced transcription and production of miR-31. Intriguingly, these transcription factors are all known targets of JNK and p38, of which c-Jun can be activated by JNK MAPK, while c-Fos and GATA2 can be activated by p38 MAPK [16–20]. This is consistent with our finding that inhibiting p38 and JNK impairs the transcription of miR-31 in response to IL-1β. It has been reported that AP1 (heterodimer of c-Jun and c-Fos) and GATA2 may act cooperatively in regulating gene transcription, which is in line with our data [23]. It is worth mentioning that both p38 and JNK have also been shown to modulate the transcription of E-selectin [13], a target of miR-31. Recent mathematical modeling and single-cell reporter assays show that the key function of miRNAs is to regulate the “noise”, or variability, of protein expression, in order to confer precision and stability to protein expression [24]. In this regard, in physiological conditions, JNK and p38 may be of pivotal importance in the expression and the fine-tuning of E-selectin, by mediating its transcription and controlling its translation through miR-31 regulation.

In accordance with our finding that miR-31 regulates the expression of E-selectin, inhibiting miR-31 is associated with increased adhesion and transendothelial migration of colon cancer cells to, and through, endothelial layer, both of which are abolished by E-selectin neutralizing antibody. These findings support our argument that miR-31 has anti-metastatic properties against colon cancers. MiR-31 has already been shown to have anti-metastatic properties for breast cancers via targeting GNA13 and WAVE3 [25, 26]. It has also been reported to inhibit gastric carcinogenesis and progression by targeting Smad4 and SGPP2 [27]. Alternatively, miR-31 can also reduce gastric tumor cell invasion and metastasis by targeting integrin α5 to indirectly affect the PI3K/AKT pathway of cancer cells [28]. In brief, miR-31 has previously been shown to reduce the metastatic potential of cancer cells by targeting signalling and cytoskeletal remodeling proteins in cancer cells. Our findings now show for the first time that miR-31 can also exert its anti-metastatic ability by targeting E-selectin, an adhesion molecule in endothelial cells. Hence, miR-31 may be a key player in the metastatic process of not only colorectal carcinoma, but also breast, bladder, gastric, and pancreatic carcinoma, leukemia and lymphoma, which all depend on E-selectin for their extravasation [2, 7].

In conclusion, our study highlights miR-31 as a key player regulating E-selectin-dependent colon cancer metastasis. Our findings further raise the possibility that miR-31 may be maintained at a low level in endothelial cells constantly expressing E-selectin, thus promoting TEM of cancer cells during metastasis. In this context, increasing the expression of miR-31 in endothelial cells may be envisioned as an approach to reduce metastases of cancer cells which extravasate in an E-selectin-dependent manner into organs as various as liver, bone marrow, skin, and lung [2, 7]. In corollary, it might be expected that a low level of miR-31 in endothelial cells could serve as a biomarker of pro-metastatic states.

## MATERIALS AND METHODS

### Reagents and antibodies

PD098059, LY294002, SP600125 and SB203580 chemical inihbitors were obtained from Sigma (St Louis, MO). Calcein-AM was obtained from Invitrogen Molecular Probes (Burlington, ON). Dimethylsulfoxyde was purchased from Fisher (Montreal, QC). IL-1β was obtained from R&D Systems (Minneapolis, MN). Anti-phospho-ERK1/2 MAPK (T^202^/Y^204^) mouse antibody, anti-phospho-Akt (S^473^) rabbit antibody, anti-Akt rabbit antibody, anti-phospho-c-Jun (S^63^) rabbit antibody, anti-c-Jun rabbit antibody were obtained from Cell Signaling Technology (Beverly, MA). Anti-E-selectin mouse monoclonal antibody was obtained from R&D Systems (Minneapolis, MN). Anti-GAPDH mouse antibody was obtained from Novus Biologicals (Oakville, ON). Anti-actin rabbit antibody was obtained from Sigma (St Louis, MO). Anti-ERK1/2 rabbit antibody, anti-phospho-HSP27 rabbit antibody, anti-HSP27 rabbit antibody were kind gifts from Dr. Jacques Landry (CRCHU de Québec-Université Laval, QC). Anti-mouse/rabbit-IgG-horseradish-peroxidase (HRP) goat antibodies were obtained from The Jackson Laboratory (Bar Harbor, ME).

### Cells

Human umbilical vein endothelial cells (HUVECs) were isolated by collagenase digestion of umbilical veins from undamaged fresh cords, as described [29]. The cords were obtained under protocols approved by the CRCHU de Québec-Université Laval Ethic Committee. HUVECs at passages ≤5 were grown to form monolayer in EGM2 endothelial cell growth medium (Lonza, Allendale, NJ) in gelatin-coated tissue culture flasks. Human liver sinusoidal microvascular endothelial cells (HLSMECs, Cell Systems, WA) were grown to form monolayer in CSC complete medium (Cell Systems, WA) in gelatin-coated tissue culture flasks.

HT29 (ATCC) colorectal adenocarcinoma cells were cultivated in McCoy 5A medium (Sigma, St Louis, MO) supplemented with 10% foetal bovine serum (FBS). HEK293T human embryonic kidney cells (ATCC) were cultivated in DMEM (Lonza, Allendale, NJ) supplemented with 10% FBS. LoVo (ATCC) colorectal adenocarcinoma cells were cultivated in Ham's F12 nutrient mixture (Life Technologies, Carlsbad, CA) supplemented with 10% FBS.

All cells were cultivated at 37°C in 5% CO_2_ humidified atmosphere.

### Plasmids, shRNAs, miRNA mimics and inhibitors

MiRIDIAN microRNA inhibitors were obtained from Dharmacon (Lafayette, CO, USA). These antimiRs bind to microRNAs by complementary sequences and thus block their capacity to silence mRNA target without affecting the level of microRNAs [30]. PsiCHECK2 vectors containing wild type SELE 3′UTR was a kind gift from Dr. Yajaira Suarez (Yale School of Medicine, New Haven, CT). This vector has a *Renilla* luciferase open reading frame upstream of E-selectin 3′UTR, and a constitutively expressed firefly luciferase open reading frame. All constructs were confirmed by sequencing. Lentiviral particles containing shRNAs against c-Jun (TRCN0000039589, TRCN0000039590), c-Fos (TRCN0000016004, TRCN000016007) and GATA2 (TRCN0000019264, TRCN0000019265) were obtained from Dr. Stéphane Gobeil (CRCHU de Québec-Université Laval, QC).

### Transfection and infection

Endothelial cells were transfected using X-tremeGene HP Transfection Reagent following manufacturers' protocol (Roche Life Science, Laval, QC). HEK293T cells were transfected with calcium-phosphate-mediated transfection method with HEPES buffered saline of Sigma (St Louis, MO). Endothelial cells were infected by lentivirus in the presence of 8 μg/mL hexadimethrine bromide (Sigma, St Louis, MO).

### RNA extraction and quantification

Cells were lysed in TRIzol (Invitrogen, Carlsbad, CA) to extract total RNA following manufacturer's protocol. The quality of RNA was assessed on agarose gel and spectrometry.

The reverse transcription was performed with TapMan MicroRNA Reverse Transcription Kit (Applied Biosystems, Foster City, CA) using specific primers (Life Technologies, Carlsbad, CA) for microRNAs, or random primers for pri-microRNAs. The quantification was carried out with Universal PCR Master Mix (Life Technologies, Carlsbad, CA) and specific probes from the same company.

### Western blotting

Cells were lysed using SDS-PAGE loading buffer without reducing agents. Proteins were separated by SDS-PAGE and transferred to a nitrocellulose membrane. Antibodies were applied according to their manufacturers' protocols. Blots were developed with SuperSignal West Pico Substrate (Fisher, Montreal, QC).

### Luciferase reporter assay

Previously described psiCHECK2 vectors were transfected into HEK293T cells with calcium-phosphate-mediated transfection method. After 48 hours, cells were lysed and the luciferase activity was evaluated using Dual-Luciferase Reporter Assay System (Promega, Madison, WI). Luciferase activities were measured by Luminoskan Ascent Microplate Luminometer (Thermo Scientific, Waltham, MA).

### Adhesion assay

Endothelial cells were plated on gelatin-coated wells and left to grow to confluence. HT29 cells were labeled with calcein-AM for 30 min at 37°C, then were added to IL-1β-activated endothelial cells for 30min. The endothelial layer was washed twice with PBS and the attached cells were quantified by measuring the fluorescence emission with Fluoroskan Ascent™ Microplate Fluorometer (Thermo Scientific). To study the E-selectin-dependence of the adhesion, neutralizing anti-E-selectin antibodies were introduced one hour before adding HT29 or LoVo cells.

### Transendothelial migration assay

Cell migration was investigated using a modified Boyden chamber assay. Endothelial cells were grown to confluence on a 5.0μm-pore-sized gelatinized polycarbonate membrane separating the two compartments of a 6.5mm migration chamber (Transwell, Costar, MA). After IL-1β-mediated activation of endothelial cells for 4 hours, calcein-AM stained HT29 cells suspended in migration buffer (medium199, 10mM HEPES pH7.4, 1.0mM MgCl_2_, 0.5% BSA) were added to the monolayer of endothelial cells, previously washed with the same buffer. After five hours, cells on the upper face of the membrane were scraped with a cotton swab. The number of HT29 and LoVo cells that have migrated to the lower face of the filter was counted using an inverted fluorescence microscope.

## SUPPLEMENTARY FIGURES


