# Supplementary material for: p38 and JNK pathways control E-selectin-dependent extravasation of colon cancer cells by modulating miR-31 transcription
Source: Oncotarget. 2016 Dec 2;8(1):1678–87. doi: 10.18632/oncotarget.13779 (PMC5352088; doi:10.18632/oncotarget.13779)
Supplement: Supplementary file 1 [file oncotarget-08-1678-s001.pdf]

p38 and JNK pathways control E-selectin-dependent extravasation of colon cancer cells by modulating miR-31 transcription

SUPPLEMENTARY FIGURES

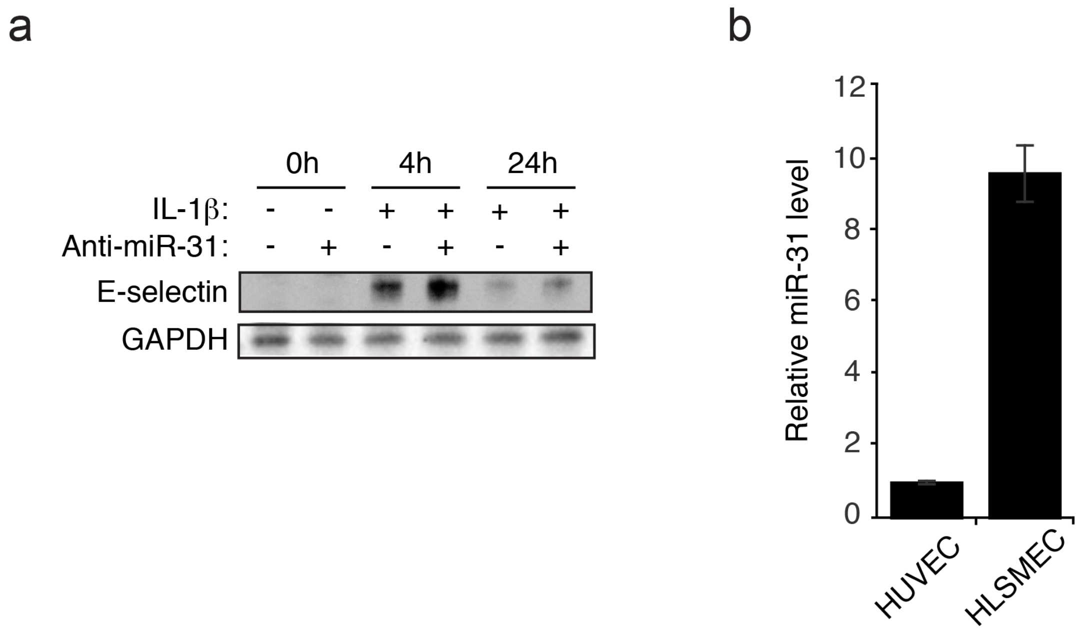

Supplementary Figure S1: miR-31 affects E-selectin level in human liver sinoidal microvascular endothelial cells. a. HLSMEC endothelial cells were transfected with 75nM of either anti-miR-31 (+) or corresponding inhibitor negative control (-) before the addition (+) of IL-1β (20ng/ml). Western blotting monitored the expression of E-selectin and endogenous GAPDH was used as loading control. The Western blot shown a representative of five independent experiments. **b.** Comparative analysis of miR-31 miRNA levels in HUVEC and HLSMEC endothelial cells. The miR-31 level was measured by quantitative reverse transcription-PCR and the snRNA U6 was used as the normalization control. The error bars represent standard errors of three independent experiments.

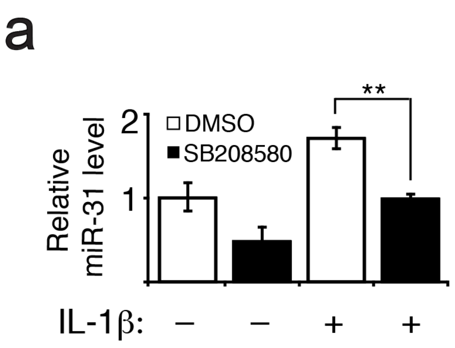

Supplementary Figure S2: IL-1β induces the production of miR-31 via p38 and JNK. The miR-31 level from samples treated with either p38 inhibitor **a.** or JNK inbitor **b.** presented in Figure 2B-C was measured by quantitative reverse transcription-PCR and the snRNA U6 was used as the normalization control. The error bars represent standard errors of three independent experiments and p values were obtained using Student's t-test (**p<0.01).

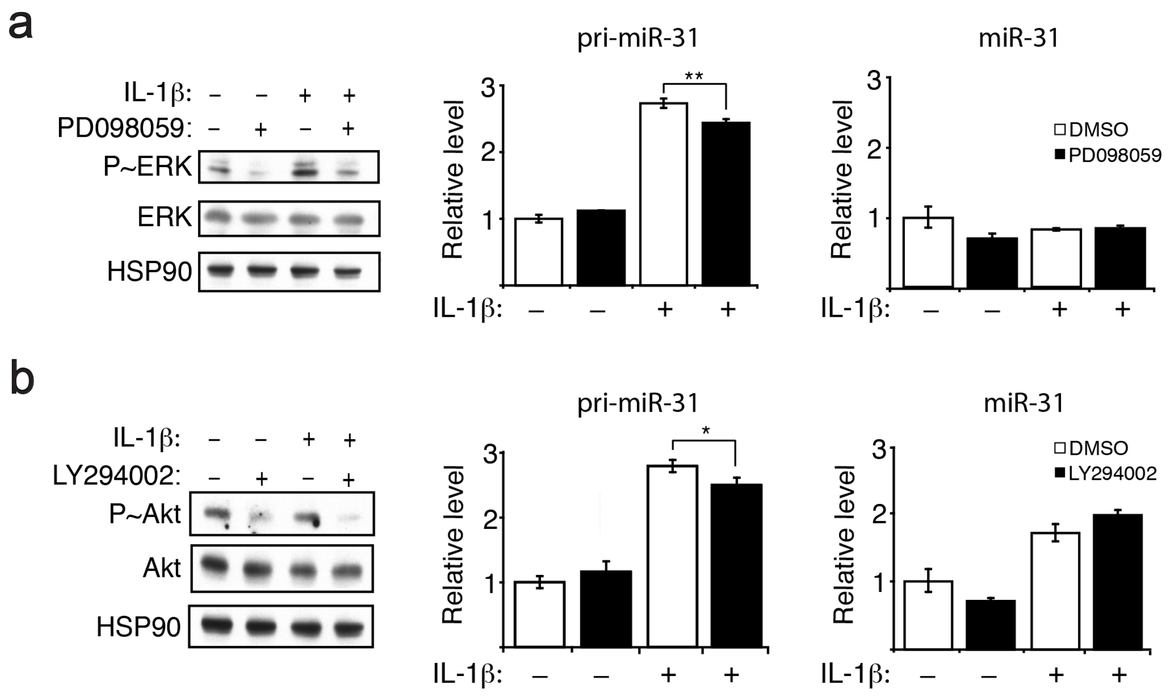

Supplementary Figure S3: The activation of the ERK and Akt signaling pathways by IL-1β do not affect miR-31 expression. Endothelial cells were pre-treated with either control DMSO or 50µM of ERK inhibitor PD098059 **a.** or 5µM of Akt inhibitor LY294002 **b.** for 1 hour before the addition of IL-1β (20ng/ml). The inhibition of those signalling pathways was confirmed by Western blotting monitoring phospho-ERK (P~ERK) and phospho-Akt (P~Akt), as indicated. The levels of primary miR-31 (pri-miR-31) and miR-31 relative to GAPDH mRNA and snRNA U6 respectively, were determined by quantitative reverse transcription-PCR. The error bars represent standard errors of four independent experiments and p values were obtained using a Student's t-test (*p <0.05; **p<0.01).

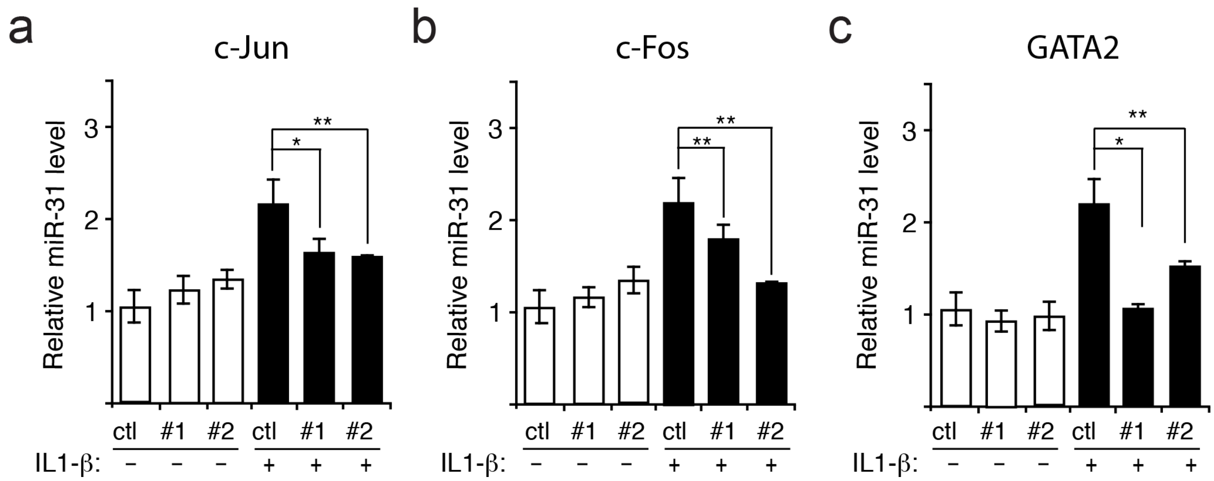

Supplementary Figure S4: IL-1β induces the transcription of miR-31 via c-Jun, c-Fos and GATA2. The miR-31 level from samples upon shRNA knockdown targeting c-Jun **a.**, c-Fos **b.** and GATA2 **c.** presented in Figure 3 was measured by quantitative real-time PCR and the snRNA U6 was used as the normalization control. The error bars represent standard errors of three independent experiments and p values were obtained using a Student's t-test (*p <0.05; **p<0.01).
